# Assessing Viral Abundance and Community Composition in Four Contrasting Regions of the Southern Ocean

**DOI:** 10.3390/life10070107

**Published:** 2020-07-05

**Authors:** Ana Sotomayor-Garcia, Maria Montserrat Sala, Isabel Ferrera, Marta Estrada, Evaristo Vázquez-Domínguez, Mikhail Emelianov, Pau Cortés, Cèlia Marrasé, Eva Ortega-Retuerta, Sdena Nunes, Yaiza M. Castillo, Maria Serrano Cuerva, Marta Sebastián, Manuel Dall’Osto, Rafel Simó, Dolors Vaqué

**Affiliations:** 1 Institut de Ciències del Mar-Consejo Superior de Investigaciones Científicas (ICM-CSIC), Passeig Marítim de la Barceloneta, 37-49, 08003 Barcelona, Catalonia, Spain; msala@icm.csic.es (M.M.S.); marta@icm.csic.es (M.E.); Mikhail@icm.csic.es (M.E.); paucortes@icm.csic.es (P.C.); celia@icm.csic.es (C.M.); yaiza@icm.csic.es (Y.M.C.); mserracu@gmail.com (M.S.C.); msebastian@icm.csic.es (M.S.); dallosto@icm.csic.es (M.D.); rsimo@icm.csic.es (R.S.); 2 Instituto Español de Oceanografía (IEO), Centro Oceanográfico de Málaga, 29640 Fuengirola, Spain; isabel.ferrera@ieo.es; 3 C/Condi de la Cañada s/n, 10857 Acebo (Cáceres), Spain; ristovazquez@gmail.com; 4 Laboratoire d’Océanographie Microbienne, (CNRS UMR7621), 66650 Banyuls sur Mer, France; ortegaretuerta@obs-banyuls.fr; 5 Red Sea Research Center (RSRC), King Abdullah University of Science and Technology (KAUST), Thuwal 23955, Saudi Arabia; sdena923@hotmail.com; 6 Institute of Oceanography and Global Change (IOCAG), University of Las Palmas de Gran Canaria (ULPGC), 35214 Telde, Spain

**Keywords:** viral abundance, viral community composition, prokaryotes, phytoplankton, environmental variables, secondary metabolic compounds, Southern Ocean, Antarctic Ocean, Antarctic Peninsula

## Abstract

We explored how changes of viral abundance and community composition among four contrasting regions in the Southern Ocean relied on physicochemical and microbiological traits. During January–February 2015, we visited areas north and south of the South Orkney Islands (NSO and SSO) characterized by low temperature and salinity and high inorganic nutrient concentration, north of South Georgia Island (NSG) and west of Anvers Island (WA), which have relatively higher temperatures and lower inorganic nutrient concentrations. Surface viral abundance (VA) was highest in NSG (21.50 ± 10.70 × 10^6^ viruses mL^−1^) and lowest in SSO (2.96 ± 1.48 × 10^6^ viruses mL^−1^). VA was positively correlated with temperature, prokaryote abundance and prokaryotic heterotrophic production, chlorophyll a, diatoms, haptophytes, fluorescent organic matter, and isoprene concentration, and was negatively correlated with inorganic nutrients (NO_3−_, SiO_4_^2−^, PO_4_^3−^), and dimethyl sulfide (DMS) concentrations. Viral communities determined by randomly amplified polymorphic DNA–polymerase chain reaction (RAPD-PCR) were grouped according to the sampling location, being more similar within them than among regions. The first two axes of a canonical correspondence analysis, including physicochemical (temperature, salinity, inorganic nutrients—NO_3−_, SiO_4_^2−^, and dimethyl sulfoniopropionate -DMSP- and isoprene concentrations) and microbiological (chlorophyll a, haptophytes and diatom, and prokaryote abundance and prokaryotic heterotrophic production) factors accounted for 62.9% of the variance. The first axis, temperature-related, accounted for 33.8%; the second one, salinity-related, accounted for 29.1%. Thus, different environmental situations likely select different hosts for viruses, leading to distinct viral communities.

## 1. Introduction

Marine viruses, the smallest and most abundant biological entities in the sea, play key roles in biogeochemical cycles, shape microbial communities, and are the largest reservoir of diversity throughout the water column from the tropics to polar systems [1,2]. The Southern Ocean (SO), which surrounds Antarctica, plays an essential role in regulating the world’s climate by contributing to the global water circulation system. One of its main traits is the Antarctic Circumpolar Current (ACC), which flows clockwise around Antarctica. The SO is characterized by low temperature and relatively low salinity waters and comprises open sea regions in which high inorganic nutrient availability coexists with low chlorophyll concentrations (HNLC regions), primarily due to a lack of iron supply for phytoplankton to perform photosynthesis [3] and the limited light during most of year. The phenomenon of lower algal biomass than that expected for the high concentrations of inorganic nutrients present in the system is known as the Antarctic paradox [4]. However, during the Austral summer, notable phytoplankton blooms occur throughout the area [5,6]. These are followed by the proliferation of prokaryotes, heterotrophic protists and viruses, which can reach high levels of abundance, activity, and diversity [7]. Processes like grazing by protists and/or zooplankton (through sloppy feeding), as well as viral lysis of prokaryotes and phytoplankton, break microbial cells [8] and promote the leaching of organic matter and micronutrients, such as iron-rich organic compounds, which become available for the growth of prokaryotic and eukaryotic phytoplankton [9,10,11,12].

Furthermore, viruses, phytoplankton, and prokaryotes are presumably key agents involved in making secondary metabolites, including volatiles that escape to the atmosphere and eventually may evolve into marine secondary aerosols, crucial in the creation of cloud condensation nuclei and, therefore, having consequent effects on climate [13,14]. A conspicuous secondary metabolite is dimethyl sulfoniopropionate (DMSP), an algal osmolyte that is produced in high intracellular concentrations by many phytoplankton taxa [15,16,17]. Two of the major aerosol-forming volatiles are isoprene (2-methyl-1,3-butadiene), which is a by-product of algal photosynthesis [14,18], and dimethyl sulfide (DMS), which derives from DMSP through the action of enzymatic lyase activity [15,16,19,20]. Generally, haptophytes, cryptophytic, and small dinoflagellates are the groups recognized to be greater DMSP producers [16,21]. DMSP is released from the cell mainly through senescence or exudation in late phases of blooms [22,23], but most importantly through grazing [20,24] and viral attack [25,26,27] and is partly transformed into DMS. Isoprene is more related to photosynthetic activity as its concentration peaks match those of phytoplankton activity [28,29,30,31], and chlorophyll-normalized isoprene production rates are known for their different phytoplankton taxa (see Booge [32] for a review). However, the relationship between phytoplankton, viruses, and isoprene remains poorly explored.

The dominating groups of phytoplankton during austral summer are diatoms, dinoflagellates, haptophytes, cryptophytes, and prasinophytes [33,34,35,36]. Phytoplankton proliferations are followed by specific prokaryotic taxa [37] that covary with changes in the phytoplankton community [38,39,40]. Both prokaryotes and phytoplankton are subjected to grazing by protists and lysis by viruses [41,42,43,44]. Environmental fluctuations could influence the abundance and activity of potential hosts, which will imply changes in the abundance and composition of the viral community. This was observed in several Antarctic lakes ([45] and refs therein), where changes from a single-stranded DNA to a double-stranded DNA–virus-dominated assemblage appeared to respond to a seasonal shift in host organisms [46]. At the same time, virus mortality processes are constrained by environmental factors [47,48]. For example, it has been reported that virus-host interactions are affected by changes in temperature and salinity [49,50], as well as by inorganic and organic nutrient concentrations, as viruses have a high demand for nitrogen and phosphorous during replication [51]. However, for Antarctic marine waters, there is still little information known on the sources of viral abundance and community variation, except for the study carried out by Miranda [52] who described seasonal changes of ssRNA viral communities in coastal Antarctic waters, in relation to variations of phytoplankton communities in spring and summer.

In this study, our goal was to explore the relationships between physicochemical and biological factors, and the abundance and composition of marine viral communities from the surface and the DCM (deep chlorophyll maximum). We studied these relationships in four regions of the Southern Ocean, characterized by distinct hydrographic conditions and dominated by different phytoplankton groups. Specifically, we tested whether (i) viral abundance and community composition were different among the four regions, and (ii) to which degree these dissimilarities were linked to the variability of potential hosts or environmental factors. To achieve our objective, we assessed the abundance and viral community composition in these four regions, and examined their potential relation to an array of (1) physicochemical (temperature, salinity, inorganic nutrients, fluorescent dissolved organic matter -FDOM- as an indicator of organic matter, and DMSP, DMS, and isoprene concentrations), and (2) biological (prokaryote abundance and prokaryotic heterotrophic production, chlorophyll a concentration, and phytoplankton taxonomic composition and biomass) variables. Additionally, we discuss the potential role of the distinct viral communities detected at each site in the production of aerosol precursor compounds.

## 2. Materials and Methods

### 2.1. Sampling Regions and Strategy

**Figure 1:**
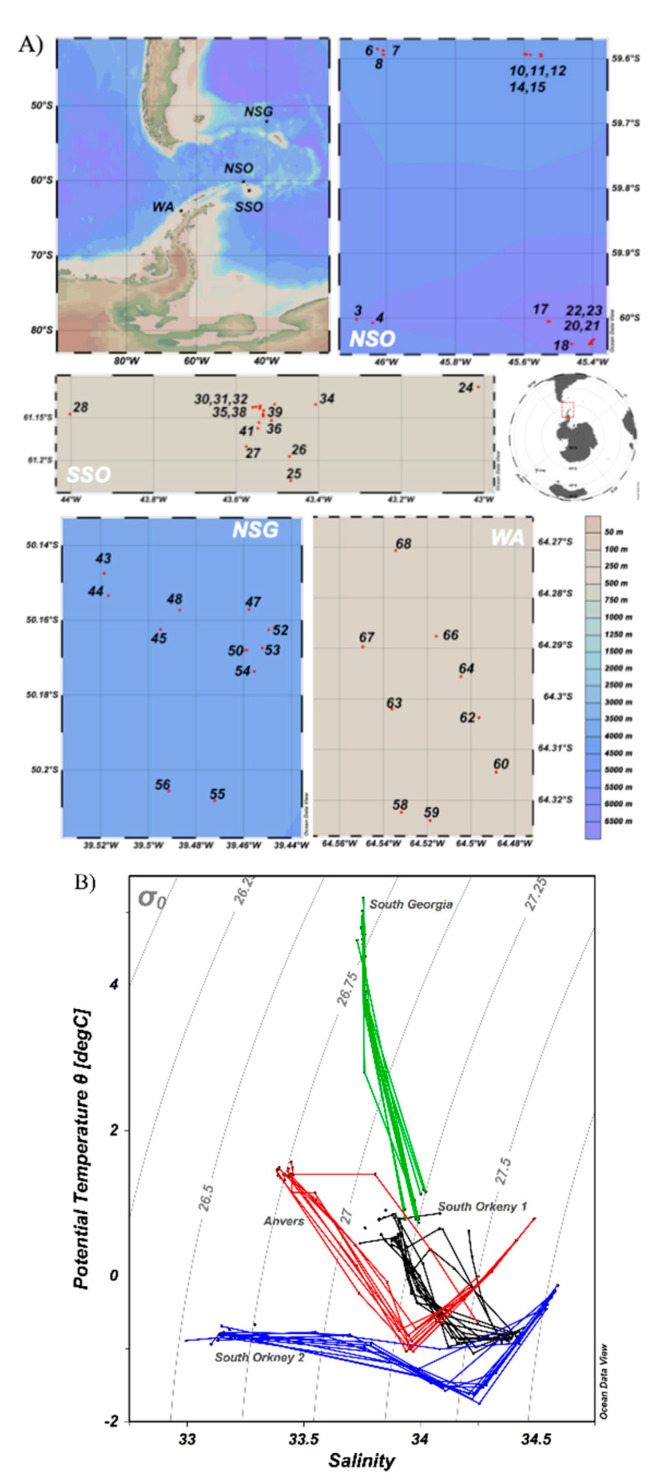
(**A**) Map of the location of the study regions: north of South Orkney, NSO; south of South Orkney, SSO; north of South Georgia, NSG; west of Anvers, WA. For each region, a zoomed window shows the position and number of the stations sampled. (**B**) Salinity–temperature diagram for the different sampled regions.

The PEGASO cruise was carried out between January and early February 2015 on board of the R/V BIO-Hespérides in the Southern Ocean near the Antarctic Peninsula, and the Sub-Antarctic Ocean (Figure 1A). Four zones were chosen for a several-day study following a Lagrangian approach: north of the South Orkney Islands (NSO), southeast of the South Orkney Island (SSO), northwest of South Georgia Island (NSG), and west of Anvers Island (WA). The position of the main hydrographic fronts along the cruise was determined following the scheme of Orsi et al. (1995) [53] (see Nunes et al. [36] for more details), with reference to the continuous records of temperature and salinity (thermosalinograph SBE 21 SeaCAT). Current velocity and direction were measured with the Shipboard Acoustic Doppler Current Profiler (SADCP) “Ocean Surveyor” at 75 khz, and the synoptic modeling data were obtained from the Global Real-Time Forecast System (Global RTOFS) [54]. In addition, we used 8-day average satellite images of chlorophyll-a concentration and sea surface temperature obtained from the Visible and Infrared Scanner (VIRS), NASA. In three of the zones (NSO, NSG, and WA), the studied water bodies were marked using WOCE (World Ocean Circulation Experiment) standard drifters provided with the Iridium communication system; in SSO, icebergs were used as Lagrangian markers. Seawater samples were collected at least once a day, at around 8:30 solar time (local time), with 12 L PVC Niskin bottles attached to a rosette sampling system. Samples were collected at several depths, from the surface (4 m) to 200 m; however, for the present study we used the 4 m and the DCM depth samples. A total of 50 stations were sampled for our purposes (16 at NSO, 14 at SSO, 11 at NSG, and 9 at WA, Figure 1A, and Tables S1 and S2).

### 2.2. Physicochemical Variables

Profiles of salinity, temperature, and fluorescence were obtained with a CTD probe (SeaBird 911 Plus) attached to the rosette. The CTD cast was deployed down to 200 m deep at all stations. Inorganic nutrients, fluorescent dissolved organic matter (FDOM), dimethyl sulfoniopropionate (DMSP), dimethyl sulfide (DMS), and isoprene concentrations were sampled at all stations, but only assessed at the surface (4 m). For inorganic nutrient (nitrate (NO_3_^−^), nitrite (NO_2_^−^), and ammonium (NH_4_^+^), silicate (SiO_4_^2−^), and phosphate, P (PO_4_^3−^)) concentrations, samples of 10 mL were collected from all stations and kept frozen (−20 °C) until analyzed in the laboratory of the Institut de Ciències del Mar, CSIC (ICM-CSIC) in an auto-analyzer (Bran + Luebbe AA3), following standard spectrophotometric methods [55]. For FDOM, we collected 250 mL sample in acid-cleaned glass bottles. The water sample was filtered through 0.2 µm acid-cleaned polycarbonate filters and analyzed onboard using a Perkin Elmer LS55 luminescence spectrometer. Single measurements for all surface samples were carried out in a 1 cm acid-cleaned quartz fluorescence cell at a constant room temperature and rinsed with a filtered water sample before analyses. We used the excitation/emission (Ex/Em) of 280/350 nm wavelengths to determine FDOM peak-T as an indicator of protein-like substances and of labile DOM [56,57]. Fluorescence measurements were expressed in Raman Units (R.U.), following Lawaetz and Stedmon [58], by normalization to the integrated water Raman scattering band of Milli-Q water freshly generated onboard every day. For DMSP analysis, two pellets of NaOH were added to 30 mL surface samples in gas-tight glass vials for 24-h hydrolysis to volatile DMS. Once in the ICM-CSIC lab, aliquots of 0.1 to 1 mL were injected into a purge flask with 2 mL of high purity water and purged for 4–6 min with ultrapure Helium. Volatiles were trapped in a Teflon tube loop submerged in liquid nitrogen, from where they were revolatilized by dipping the loop in hot water and injecting it into a gas chromatography system (GC-14A, Shimadzu). Sulfur compounds were separated using a packed CarbopackH 60/80 mesh column (Sigma-Aldrich, St. Louis, MO, USA) maintained at 170 °C and detected with a flame photometric detector. Retention time for DMS was 0.9 min, and the detection limit was 3 pmol. Analytical precision was better than 5%. Calibration was performed by syringe injection into the purge vial of varying volumes of a gaseous mixture of He and DMS released by a weight-calibrated permeation tube (Dynacal, Valco Instruments Co. Inc., Houston, TX, USA). DMSP concentrations were calculated by subtraction of the endogenous DMS. Isoprene and DMS were determined, along with other volatiles, on a gas chromatography-mass spectrometry system (5975-T LTM GC/MS, Agilent Technologies, Santa Clara, CA, USA). Aliquots of 25 mL were drawn from the glass bottle with a glass syringe with a Teflon tube and filtered through a 25 mm glass fiber filter while being introduced into a purge and trap system (Stratum, Tekmar Teledyne, Mason, OH, USA). Volatiles were stripped by bubbling with 40 mL min^−1^ of ultrapure He for 12 min, trapped on solid adsorbent (VOCARB 3000) at room temperature, and thermally desorbed (250 °C) into the GC. DMS and isoprene, monitored as *m*/*z* 62 and 67, respectively, in selected ion monitoring mode, had retention times of 2.4 and 2.5 min in the LTM DB-VRX chromatographic column held at 35 °C. The detection limit was 0.01 pmol (0.5 pmol L^−1^ in the samples), and the median analytical precision was 5%. DMS calibration was performed with DMS solutions generated by dissolution and hydrolysis of solid DMSP (TCI, Tokyo, Japan) in high purity water. Calibration of isoprene was performed by injections of a gaseous mixture of isoprene in N_2_ (provided by the University of California Irvine).

### 2.3. Microbiological Variables

The biomass of phytoplankton, abundances of viruses and prokaryotes, and prokaryotic heterotrophic production, were assessed in all stations (surface and DCM), while viral communities’ compositions were estimated in 20 stations at the surface and 17 at the DCM (Table S2). Chlorophyll a concentration (Chl-a) was determined on board in 250 mL sub-samples that were filtered through glass-fiber filters (Whatman GFF). This filtration was followed by extraction with 90% acetone of at least 24 h in the dark at 4 °C. The fluorescence of the extracts was measured with a calibrated fluorometer (Turner Designs), and no phaeophytin corrections were applied [59]. Microalgae were identified and quantified by pigment composition determined by HPLC [60]. CHEMTAX (chemical taxonomy software, version 1.95) was used to derive the contribution of microalgal groups for the total Chl-a biomass from pigment data [61]. Seven pigmentary classes were quantified: chlorophytes, cryptophytes, diatoms, dinoflagellates, haptophytes, prasynophytes, and pelagophytes (see Nunes et al. [36] for details). For our purposes, we only used the three most representative groups (cryptophytes, haptophytes, and diatoms) that each contributed with more than 20% each to the total chlorophyll a concentration in the visited areas. Sub-samples (2 mL) for viral abundance (VA, dsDNA-double stranded DNA- viruses) were fixed with glutaraldehyde (0.5% final concentration), flash-frozen in liquid nitrogen, and stored at −80 °C. Once in the ICM-CSIC laboratory, these sub-samples were thawed and stained with SYBR Green I before counting [62]. Groups of viruses were determined in bivariate scatter plots of green fluorescence of stained nucleic acids versus side scatter [42]. Viruses were classified as low (V1), medium (V2), and high (V3) according to fluorescence signal. Presumably, the V1 and V2 fractions are mainly attributed to bacteriophages, although some eukaryotic algal viruses may display low fluorescence, and V3 to phytoplankton viruses [42]. Prokaryote abundance (PA) was determined in 2 mL sub-samples preserved with a 1% paraformaldehyde +0.05% glutaraldehyde solution, flash-frozen in liquid N_2_, and stored at −80 °C until analysis. Once in the ICM-CSIC laboratory, samples were stained with SYTO13 (SYTO^TM^13, ThermoFisher) and counted following the protocol of Gasol and Del Giorgio [63]. Counts of viruses and prokaryotes were carried out in a flow cytometer (FACSCalibur, Becton & Dickinson). Samples for prokaryotes were run with fluorescent yellow-green latex beads (0.92-μm) as an internal standard, and for viral counts using a flow rate of ~60 µL min^−1^. Prokaryotic heterotrophic production (PHP) was estimated by the radioactive ^3^H-leucine incorporation technique [64], according to the modifications established for the use of microcentrifuge [65]. Leucine incorporation was assessed on board in a scintillation counter (Beckman). Then,

PHP = Leu × CF (μgC L^−1^ d^−1^), (1)

where Leu is the ^3^H-leucine incorporation (pmol L^−1^ h^−1^), and CF is the conversion factor used: 1.5 kg C mol Leu^−1^ (cf. [66]).

### 2.4. Viral Community Composition: Random Amplified Polymorphic DNA (RAPD)

The randomly amplified polymorphic DNA-polymerase chain reaction (RAPD-PCR) is a practical and efficient method for measuring dsDNA viral diversity, providing viral assemblage comparisons through banding patterns. By gathering the required DNA concentration, we ensure that the analyses will give us a representative image of the original richness of the area and that the evenness distribution between samples will only respond to the initial diversity of the sample. Seawater samples (5L) from representative selected stations of the different regions and from two depths—surface and DCM (Table S2)—were sequentially filtered by 0.8 µm and 0.2 µm. Then, the 0.2 µm filtered samples were concentrated up to 50 mL with tangential flow filtration by using a spiral-wound cartridge (30-KDa VIVAFlow 200). Concentrates were stored at 4 °C until analysis. Once in the laboratory of the ICM-CSIC, the 50 mL concentrates were prefiltered through a polycarbonate filter (0.2 µm nucleopore) to eliminate any remaining prokaryote that would add prokaryotic DNA to the viral banding pattern, and ultra-concentrate by centrifuging at 4000 rpm for 10 min in 30-kDa ultra centrifugal filter tubes (Amicon, Sigma) [67]. Then, the viral ultra-concentrates were placed into agarose plug-like molding pieces. Before amplification of the viral DNA, the absence of the 16S rRNA prokaryotic gene was checked via PCR to ensure that each sample was prokaryotic-DNA free and, if it was not the case, it was treated with DNAse and further rechecked. Then, proteinase K was added in order to release the viral DNA from the protein capsid, and afterwards, treated with a cleaning protocol (Pefabloc^®^, Sigma), to inactivate this proteinase, as it can act as a Taq–polymerase inhibitor. Randomly amplified polymorphic DNA-polymerase chain reaction (RAPD–PCR) was designed (10′ at 94 °C, 30 times 3′ at 35 °C, 1′ at 72 °C and 30 s at 94 °C, 3′ at 35 °C, and 10′ at 72 °C) to amplify random sequences of the viral DNA by using the CRA22 primer (5′-CCG CAG CCA A- 3′). This primer was chosen due to its better results during the optimization of the protocol [68]. Consecutive RAPD-PCR reactions were done until 450 ng of DNA were obtained from each sample. DNA quantification was performed using a QuBit fluorometer. After that, the viral community composition was determined by using a gel electrophoresis technique. Briefly, the gel characteristics and running settings were 1.8% AG-2 at 70 Volts during 120 min and DNA was stained with 7 μL of SYBR Safe (10000X concentrate in DMSO). Finally, a UV image was taken from every gel with the gel imaging system ChemiDocTM (Bio-Rad) with a time exposure of around 2.1 s. Gel band pattern analysis was performed using the Quantity One 4.5.2 software (Bio-Rad). To make the band-distribution comparable between two different gels, before to final results we conducted banding pattern calibrations. Moreover, in the final gels, we ran replicates of some samples between gels in order to be able to couple and adjust them according to the duplicates and the ladder. Comparison of bands (operational taxonomic units, OTU) between lanes was performed by hand. Information was converted into a binary matrix representing the presence-absence of viral OTU; only communities present at more than two stations were considered in the analyses. The banding pattern analysis, i.e., the diversity analysis, was conducted with all samples, surface, and DCM, pooled together, so that we could identify which viral populations were present in both the surface and DCM.

### 2.5. Data Analyses

The map of the study regions, the temperature-salinity (T-S) diagram, and the temperature and salinity profiles were plotted using the software package Ocean Data View (ODV) [69]. Normality distribution of environmental and biological data (except for algal biomass) was checked with the Shapiro–Wilk test and, when necessary, continuous variables were logarithmically transformed prior to analyses to fulfill the requirements of statistical tests. The mean value of all variables was calculated considering all measurements performed at surface (n = 47–50) and at the DCM (n = 43–44). Spearman correlation analyses were used to determine the relationship between viral (total and V1-V3) abundances and biological and environmental variables. The Bonferroni–Holm correction was applied to each set of correlation coefficients. Multiple regression analysis was used to assess the predictor variables for the isoprene metabolite concentration. This was performed with the lm function from the R program. The best models are considered to be those that maximize the R^2^ and minimize the standard error of the estimate, with the minimum number of predictive variables. The distribution of abundances and concentrations of the biological variables and the analyses of the band pattern obtained with gel electrophoresis (see Section 2.4 for a detailed description of the technique) were plotted using the KaleidaGraph program. Distribution of viral community composition (i.e., similarity between band pattern among samples and sites) were plotted with non-metric multidimensional scaling plots (NMDS) using the metaMDS function in the R program. The dissimilarity matrix calculated using the Jaccard distance index and the stress factor is the statistical scale that indicates how good the 2-D representation of the original data is. Finally, the relationship between viral community composition and selected environmental variables was explored using constrained (or canonical) correspondence analyses (CCA), a multivariate ordination technique [70] in which the inertia can be interpreted as the explained variance. The group of environmental variables included in the CCA was chosen according to a step-wise selection, in a backward direction, with an AIC of 96.641. The CCA shows how the selected variables (represented as arrows) contribute to the structure of marine viral communities (represented as points colored according to the sampling zone). The lengths of the arrows are equivalent to their influence on the represented variables and exert in the viral communities (the longer the arrow, the stronger the influence). All statistical tests were performed using the vegan package from R [71].

## 3. Results

### 3.1. Characterization of the Study Area: Environmental and Biological Variables

#### 3.1.1. Temperature and Salinity

The T–S diagram of the sampled stations revealed hydrographic differences among the visited regions (Figure 1B); this is also reflected in the vertical temperature and salinity profiles shown in Figure S1A,B. The main hydrographic fronts during the PEGASO cruise are described in Nunes et al. [36], following Orsi et al. [53]. The NSO and NSG regions were located within meanders of the southern boundary of the eastwards Antarctic circumpolar current (SBACC) and the polar front (PF). SSO was nearly 60 nautical miles north of the Weddell front (WF) and next to the marginal ice zone of the Weddell Sea in winter. WA was also placed on the southern boundary of the Antarctic circumpolar current (SBACC). NSG had the warmest waters, reaching 4.8 °C at the surface, and presented rather homogeneous salinity profiles (around 33.80, Figure S1A). NSO and SSO, in spite of their proximity, had different mean temperatures (seasonally warmed) and salinity (ice-melt water influenced), meaning SSO was significantly colder and less saline than NSO (Table 1, Figure S1). Finally, WA had also low salinity, probably was also affected by ice-melt water, and intermediate water temperatures (Figure S1B). The thermocline was appreciable at around 30 m (NSO), 16 m (SSO), 50 m (NSG), and 23 m (WA) (Figure S1).

#### 3.1.2. Inorganic Nutrients, FDOM and Secondary Compounds

Mean concentrations of inorganic nutrients (NO_3_^−^, NO_2_^−^, NH_4_^+^, SiO_4_^2−^, PO_4_^3−^) and fluorescent dissolved organic matter (FDOM–peakT), as well as secondary metabolites (DMSP, DMS, and isoprene) measured at the surface, varied among sampling regions (Table 1). Nitrite and ammonia had almost negligible concentrations, compared to those of nitrate. Nitrate and phosphate concentration averages reached higher values in SSO and NSO (27.76–28.44 µM NO_3_^−^ and 2.04–2.15 µM PO_4_^3−^, respectively) than in NSG and WA (18.35–18.63 µM NO_3−_, and 1.42–1.81 µM PO_4_^3−^, respectively). Then, silicate showed extremely low average values at NSG (mean: 2.02 ± 20.73 µM SiO_4_^2−^, Table 1) and very high between 45.72 and 49.75 µM at the other regions. Although we did not measure micronutrients, there is evidence from prior studies that NSG is placed in an iron-sufficient zone [36,72,73]. The FDOM-peakT was remarkably higher at WA (40.8 ± 6.3 R.U, Table 1) than in the other areas. DMSP concentration was two-fold higher at NSO than in the rest of the regions (249.1 ± 101.2 nM, Table 1) and DMS was highest at SSO (7.8 ± 1.6 nM, Table 1). Isoprene reached the highest concentration at NSG (76.5 ± 8.3 nM) and the lowest at SSO (4.7 ± 1.6 nM, Table 1).

#### 3.1.3. Chlorophyll a, Phytoplankton Taxa and Prokaryote Concentrations

**Figure 2:**
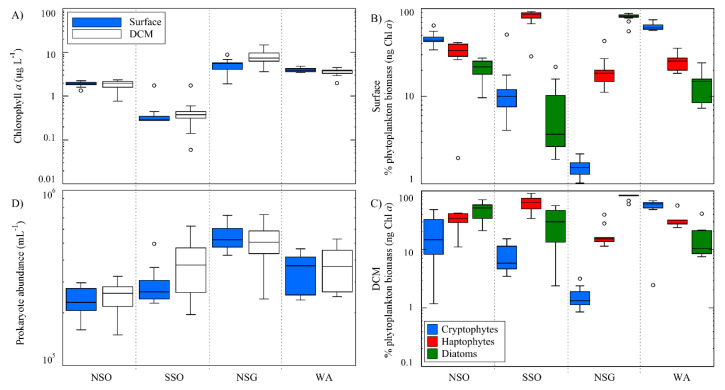
Distribution of the microbiological variables at the surface and DCM in the four visited areas. (**A**) Chlorophyll a concentration. (**B**,**C**) Percentage of haptophyte, cryptophyte, and diatom biomass, with respect to the sum of the three phytoplankton groups biomass, at the surface and at DCM, respectively. (**D**) Prokaryote abundance. See explanation in Figure 1 for acronyms.

Chl-a concentration was two-fold higher in NSG and WA than in NSO and was at nearly the limit of detection at SSO, both at the surface and at the DCM (Figure 2A, Table 1). The contribution of the dominant phytoplankton groups (cryptophytes, diatoms, and haptophytes) varied also among the sampling regions (Figure 2B,C). NSG was dominated by diatom biomass (83% of the total Chl-a-derived biomass of the three algal groups at the surface, and 85.6% at the DCM) (Table 1, Figure 2B,C). WA was dominated by cryptophytes (around 50%), followed by haptophytes (around 40%), at both depth layers (Table 1, Figure 2B,C). In NSO, the surface’s phytoplankton community showed co-dominance between cryptophytes (42.4%) and haptophytes (40.4%), with a lower presence of diatoms (17.2%), while at the DCM, diatoms and haptophytes co-dominated (44.1 and 36.7%, respectively). In the SSO, haptophytes were always dominant, reaching 78.7% of the total algal biomass (of the three groups) at the surface and 56.4% at the DCM (Table 1, Figure 2B,C). Prokaryote abundance (PA) showed the highest mean values at NSG, both at the surface and at the DCM (5.4 ± 0.9 × 10^5^ prokaryote mL^−1^, and 5.2 ± 1.0 × 10^5^ prokaryote mL^−1^, respectively (Table 1, Figure 2D)). PA differences between surface and DCM were only noticeable at SSO, where the DCM was enriched by a factor of 1.2 (Table 1, Figure 2D). Finally, PHP was highest in NSG and lowest in SSO (1.04 ± 0.44 µg C L^−1^ d^−1^ and 0.31 ± 0.18 µg C L^−1^ d^−1^, respectively (Table 1)).

### 3.2. Viral Abundance

**Figure 3:**
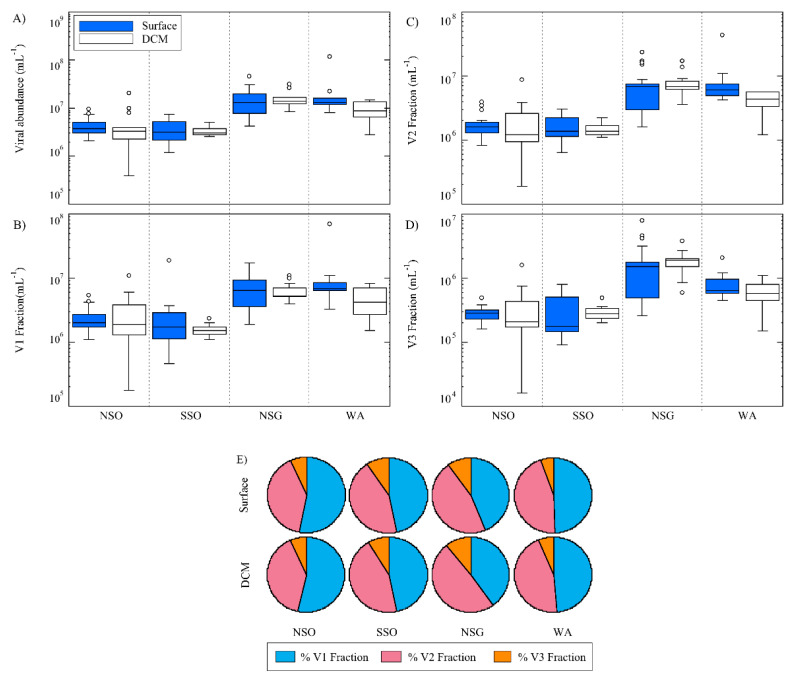
Distribution of viral abundance in the four visited areas, at the surface and DCM. (**A**) Total viral abundance. (**B**–**D**) Abundance of different viral groups (V1, V2, and V3). (**E**) Percentage of viral groups in relation to total viral abundance. Presumably, fractions of V1 and V2 are mainly attributed to bacteriophages and V3 to viruses of phytoplankton. See explanation in Figure 1 for acronyms.

Viral abundance (VA), like almost all the other variables, achieved the highest mean values at NSG (21.5 ± 10.7 × 10^6^ viruses mL^−1^ at the surface and 17.9 ± 6.6 × 10^6^ viruses mL^−1^ at the DCM (Figure 3A)) and the lowest at the surface of SSO (3.0 ± 1.5 × 10^6^ viruses mL^−1^). Mean VA generally tended to be higher at the surface than at the DCM (Table 1), except for SSO (Table 1, Figure 3A). The contributions of low and medium fluorescence viral abundances (V1 and V2) to total viral abundance (VA) were similar (Figure 3B,C,E), except for NSG, where V2 was slightly higher than V1 (Table 1, Figure 3B,E). The V3 fraction (viruses with high fluorescence content, Figure 3 D) was less abundant at all regions, reaching on average ~7%, 6%, 10%, and 6% of the total VA at NSO, SSO, NSG, and WA, respectively (Figure 3E). Viral abundance was positively correlated with temperature, PA, Chl-a, PHP, diatom and haptophyte biomass, nitrite, FDOM, and isoprene concentrations (Table 2). Conversely, VA covaried negatively with nitrate, silicate, phosphate, and DMS concentrations (Table 2).

### 3.3. Viral Community Composition

**Figure 4:**
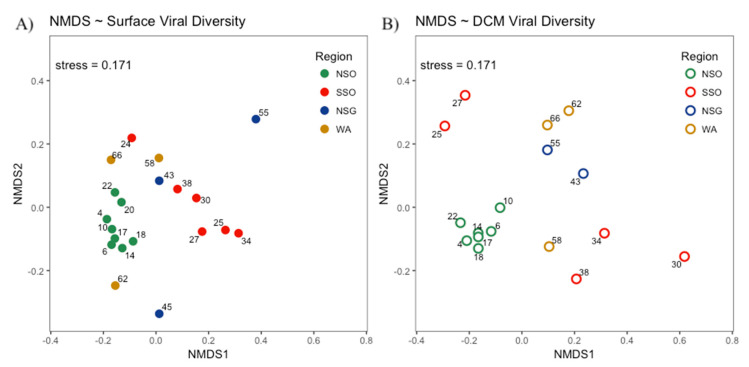
Non-metric multidimensional scaling (NMDS) plots of the marine viral community composition. (**A**) Surface viral diversity. (**B**) DCM viral diversity. Plotted data arise from a matrix of similarity drawn by the banding pattern of randomly amplified polymorphic DNA (RAPD)-PCR products obtained for the pooled set of samples of the surface and DCM (Figure S2). Full dots represent surface communities and empty circles represent DCM samples. Numbers correspond to the station sampled. See explanation in Figure 1 for acronyms.

We assessed viral diversity from 20 stations at the surface (8 in NSO, 6 in SSO, 3 in NSG, and 3 in WA) and from 17 at the DCM (7 in NSO, 5 in SSO, 2 in NSG, and 3 in WA). Considering all stations, we observed 22 distinct RAPD bands ranging in size from about 250 bp to 2000 bp (Figures S2 and S3). The banding pattern from each sample displayed in the agarose gels (Figure S2), corresponds to the most abundant populations of the viral community present at each station and gives an idea of the viral community composition. Thus, if we compare the band pattern of each lane (i.e., station), we can assess the viral diversity among and within regions and depths layers. NSG accounted for 76.7% of the detected bands, followed by NSO with 66.7%, WA with 60%, and SSO with 53.3%. From those, NSO was the region with a higher number of bands relative to the sampling effort (i.e., sampled stations per region). On average, half of the bands observed at the surface were also detected at the DCM of the same station (58% at NSO, 37% at SSO, 58% at NSG, and 61% at WA (Figure S3)). Several bands were only present at the NSO region (e.g., bands: 3, 9, 11, 14 (Figure S3)) suggesting some viral population endemism, while other bands (e.g., bands 15, 16, and 19 (Figure S3)) were found in different regions, pointing towards more cosmopolitan viral members. We grouped viral communities using a non-metric multidimensional scaling analysis (NMDS) (Figure 4), based on the RAPD’s band pattern, with a satisfactory stress factor of 0.171 (Figure 4 and Figure S4). Some viral communities were more similar within the same region than among regions, indicating that viruses differentiate regionally (Figure 4 and Figure S4). This can be especially appreciated with the viruses in the NSO, which form a compact group at both depth layers, revealing a highly homogeneous viral community, also appreciable for viruses from the surface at SSO (Figure 4A). In contrast, the viral communities of the WA and NSG regions were more widely dispersed, both at the surface and at the DCM (Figure 4A,B and Figure S4).

**Figure 5:**
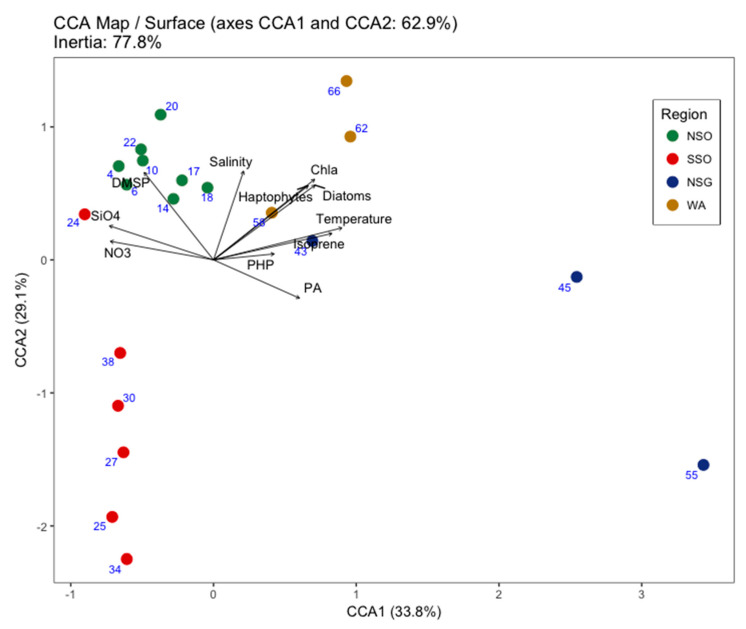
Ordination diagram displaying the canonical (or constrained) correspondence analyses (CCA). Surface marine viral communities from 20 sites, related to environmental (temperature, salinity, NO_3−_, and SiO_4_^2−^) and biological (Chl-a, PA, PHP, diatoms, and haptophytes) variables, and to secondary metabolites (DMSP and isoprene concentrations). Variables are shown as arrows, and viral community data as dots. Longer arrows denote a higher significant relationship. Same dots coloring as used in Figure 4A. Blue numbers correspond to the stations sampled. See explanation in Figure 1 for acronyms.

We used constrained correspondence analyses (CCA) (Figure 5) to explore the relationships between viral community structures and the resultant best explanatory variables according to step-wise selection. These variables were: temperature, salinity, nutrient concentrations (NO_3−_, SiO_4_^2−^), PA, PHP, Chl-*a*, and the biomass of diatoms and haptophytes. In addition, secondary metabolites (DMSP and isoprene) were considered, in order to address the role of viral communities on the gas and particle exchange for aerosolization. The first two ordination axes of the CCA accounted for 62.9% of the variance in the viral community composition with a total constrained inertia of 77.8% (Figure 5). The first ordination axis of the CCA accounting for 33.8% of the variance was mainly related to temperature on the positive side and with nutrients on the negative one. Therefore, the stations of the more temperate regions (NSG and WA, Figure S1) appeared on the right side of the graph, while those of the colder regions were found on the left side (NSO and SSO (Figure S1)). The second axis accounted for a slightly smaller proportion of the variance (29.1%) and was positively related to salinity and algae biomass (most importantly in the WA region (Figure 5)); thus, most samples of SSO, the less saline region, occupied the negative side of this axis.

## 4. Discussion

Viral communities showed some segregation differences among the sampled regions, both at surface waters and at the DCM (Figure 4). However, while some viral populations were geographically constrained, others were widely distributed, as shown by the banding pattern of the RAPD (Figures S2 and S3). Indeed, several studies have reported a high global viral diversity and almost as high local diversity [74,75,76,77,78,79], as well as connectivity along the water column [80,81,82,83]. This might be based, on one hand, in the migration capability (i.e., transport by oceanic currents and sinking attached to particles) of marine viruses [84], and on the other hand, on environment selective pressure [15,17].

Authors such as Breitbart and Rohwer [75] suggested the seed-bank theory, where high local viral diversity relies on host availability and diversity. There, viruses would move from the seed-bank state to the active phase whenever their host would be “blooming”. Once the predominant host cells decay, the empty niche would be occupied by another hosting species, and the consequent displacement of viruses from the seed-bank state would occur [75]. These ecological dynamics may apply in the present study, explaining the obtained grouping of Antarctic viral community structures according to the sampled regions (in particular in the NSO and SSO regions (Figure 4)). In addition, Brum et al. [77] proposed temperature as a relevant environmental factor that influences changes in viral activity and diversity. Thus, during the spring–summer transition, they detected a reversion from lysogenic to lytic viruses that increased the viral and microbial diversity. All these processes will favor the geographical differentiation of viral assemblages.

In our study, temperature and salinity substantially varied among regions (Figure 1B and Figure S1), and the different degrees of stratification of the water column (with mixed layers from 16 m in SSO to 50 m in NSG) might have affected the light penetration and nutrient supply from deeper layers to the upper mixed layer, as is reported in Nunes et al. [36], for the studied area. In addition, it is expected that the development of different phytoplankton species [35] and prokaryotes [82,83] may influence the environmental conditions, modifying nutrient concentration and contributing to the production of secondary metabolites, such as DMSP, DMS, and isoprene [14,15,16,17,18,85,86]. At the same time, viruses could participate in these biogeochemical processes infecting phytoplankton and prokaryotes and releasing to the environment dissolved organic matter (DOM) from the cell-enclosed material [87,88]. It makes sense, then, that our results showed a strong significant correlation between fluorescent dissolved organic matter (FDOM, peak T, considered an indicator of proteinaceous compounds [89], and labile DOM [57]), and all viral abundance fractions (Table 2). Then, viruses would intervene in regulating microbial biomass and diversity, which in turn would be reflected in the viral community composition [25,26,27,84,88]. Viral abundance related positively with that of their potential hosts (prokaryotes and phytoplankton), but negatively with some inorganic nutrient concentrations (NO_3_, SiO_4_^2−^ and PO_4_^3−^ (Table 2)). This could be a consequence of inorganic nutrient uptake by algae at rates which vary among different phytoplankton taxa and depend on micronutrient availability [36]. Thus, in the iron-rich waters of NSG [36], where diatoms dominated the phytoplankton biomass (Figure 3), the inorganic nutrient concentrations, especially silicate, registered the lowest values (Table 1). In contrast, in the other regions (NSO, SSO, and WA), where iron was limited [3], inorganic nutrient concentrations were higher (Table 1).

The potential role of viruses in producing secondary metabolites as DMSP (DMS precursor) by lysing phytoplankton host cells, was experimentally reported by Hill et al. [26]. Then, we expected that viral abundance and DMSP concentration would correlate; however, that was not reflected in our results (Table 2). Bratbak et al. [25] also observed no correlation between viral abundance and DMSP concentration and posed that bacteria degradation represents the major sink for DMSP and DMS, which is also supported by Kiene and Service [90]. Furthermore, other DMSP-freeing pathways (i.e., grazing, senescence, apoptosis, etc.) may also play a role in increasing its release [20,22,23,91,92]. In the case of DMS, its concentration was negatively correlated with viruses. DMS has been shown to be strongly dependent on microbial community composition (e.g., presence of DMS consumers [15] or demethylating bacteria [17]) and to environmental conditions (e.g., wind-related ventilation and photolysis), which modify the structure of the microbial food web [15]. This may be the case of the area south of South Orkney (SSO) area, where the highest irradiance was registered, and thus, DMSP to DMS transformation could be enhanced. The formation of isoprene is mainly attributed to the activity and physiology of phytoplankton [28,29,30,31], while the effect of viral lysis is still unclear [14,18]. In our study, although isoprene concentration and viral abundance strongly covaried (Table 2), we believe that it is not a cause–effect. We are aware that diatom viruses are mainly ssRNA and ssDNA [93], and viral abundances reported here, by FCM, refer to dsDNA. Then, through multiple regression analyses, we obtained that the model that better predicted the isoprene concentration variability highlighted diatom biomass as the only explanatory factor and excluded the dsDNA viral abundance (log isoprene conc. = −3.53 (±1.43) + 0.37(±0.07) × log diatom conc.; n = 18, R^2^ = 0.78, *p* < 0.0001). Hence, further investigation is needed in studying the interaction between diatoms and their specific viruses in the formation of isoprene. Indeed, some studies with other photosynthetic microorganisms reported an increase of isoprene production after viral infection, as is the case of *Prochlorococcus* infected by phages [14,18].

In summary, the observed regional segregation of the different viral communities (Figure 4 and Figure 5) in this study, could be a result—to a greater or lesser extent—of the presence of different potential prokaryote and phytoplankton hosts, as observed by several authors [35,50,51,77,82,83], and to the variability of some physicochemical parameters [15,17]. Viral communities at the NSO and SSO regions were associated with the coldest waters and with high inorganic nutrient concentrations (Table 1, Figure 5). In particular, the group of viruses dominating the NSO region also coexisted with high DMSP concentration (Table 1) and a high proportion of haptophyte and cryptophyte biomass (Figure 2B,C), which according to Stefels et al. [16], are the main phytoplankton taxa responsible of the DMSP production. However, this was not reflected in the increase of DMS, perhaps due to low bacterial lyase activity, important in the DMSP degradation [91,92,94], or to environmental factors (i.e., temperature and sunlight etc. [15]). In the NSG and WA regions, although the number of samples was low, viral communities were more heterogeneous. Indeed, high temperatures and the availability of different dominant potential prokaryotic and phytoplanktonic hosts may influence the viral community composition. The high prokaryotic heterotrophic production and prokaryote abundance recorded at the NSG could be the results of a high growth rate of different successful prokaryote communities, derived from the carbon released by the various diatom taxa blooming during the sampling time [36]. High isoprene concentrations can be also associated with this bloom of diatoms, according to several studies [29,95,96]. Finally, regarding the variability of viral assemblages in the WA, relatively high temperatures could be associated with an advanced state of phytoplankton succession [36], which may have been driven by the high abundance and diversity of potential hosts such as haptophytes, cryptophytes, and prokaryotes [75].

## 5. Conclusions

In the present study, we found that the Antarctic and sub-Antarctic marine viral abundances are mainly associated with temperature and the abundance and biomass of their potential hosts. Furthermore, the viral community composition showed higher similarities within areas than among regions, particularly in surface waters. Presumably, the combination of physicochemical and biological factors led to distinct scenarios, which could drive variability in marine viral communities and simultaneously influence the various components of the microbial food web. However, these relationships must be interpreted in a dynamic way, because while viruses are causing changes in prokaryote and phytoplankton communities, at the same time it is the viruses that are changing in response to shifts in prokaryotic and phytoplankton community composition. While it is known that different phytoplankton taxa are associated with distinct prokaryote communities, further characterization of prokaryotic community composition would provide more insight on this potential diversification driver. Concerning secondary metabolites, we did not find a clear relationship between viruses and DMSP, DMS, and isoprene; thus, further experimental and field studies are needed in order to disentangle the complex relationships occurring in the marine food web that are involved in their release.

## Figures and Tables

**Table 1:** Mean ± standard deviation (SD) of environmental and biological variables measured at all stations. Mean of variables only available from surface (n = 47−50 stations): nutrients: nitrate (NO_3_^−^), nitrite (NO_2_^−^), and ammonia (NH_4_^+^), silicate (SiO_4_^2−^), and phosphate (PO_4_^3-^), fluorescent dissolved organic matter (FDOM-peakT), and secondary metabolites: dimethyl sulfoniopropionate (DMSP), dimethyl sulfide (DMS) and Isoprene at the different sampling areas. Concentrations are expressed in: * µM, ** nM, and *** pM. Mean of variables available from both surface (n = 47–50) and DCM (n = 43–44): temperature, salinity, chlorophyll a concentration (Chl-a, µg L^−1^), biomasses of cryptophytes, diatoms, and haptophytes (ng Chl-a L^−1^), prokaryote abundance (PA, 10^5^ cells mL^−1^), prokaryotic heterotrophic production (PHP, µg C L^−1^ d^−1^), viral abundances (10^6^ virus mL^−1^): total (VA), low and medium (V1, V2) and high fluorescence (V3). See explanation of Figure 1 for region acronyms.

AREAS		NSO		SSO		NSG		WA	
VARIABLES	Depths	Mean	SD	Mean	SD	Mean	SD	Mean	SD
NO_3_− *	Surface	27.76	1.89	28.44	3.42	18.35	5.47	18.63	0.89
NO_2_− *	Surface	0.23	0.06	0.17	0.04	0.27	0.05	0.19	0.03
NH_4_^+^ *	Surface	1.11	3.51	1.03	1.03	0.78	2.02	2.48	1.87
SiO_4_^2−^	Surface	47.21	4.08	45.72	11.96	2.02	20.73	49.75	3.66
PO_4_^3−^ *	Surface	2.04	0.21	2.15	0.28	1.42	0.43	1.81	0.16
FDOM (R.U.)	Surface	18.5	4.9	7.9	1.3	15.4	3.1	40.8	6.3
DMSP **	Surface	249.1	101.2	103.5	57.2	84.4	28.0	115.7	14.61
DMS **	Surface	7.0	2.6	7.8	1.6	5.8	1.0	2.1	0.6
Isoprene ***	Surface	9.6	1.7	4.7	1.6	76.5	8.3	12.0	0.6
Temperature	Surface	0.5	0.3	−0.8	0.1	4.8	0.5	1.4	0.1
	DCM	0.0	0.3	−1.5	0.4	4.3	0.4	1.7	0.6
Salinity	Surface	33.88	0.073	33.14	0.064	33.74	0.007	33.41	0.002
	DCM	34.02	0.068	34.20	0.148	33.77	0.004	33.42	0.053
Chl-a	Surface	1.82	0.33	0.31	0.05	5.05	1.88	4.45	0.46
	DCM	1.61	0.38	0.37	0.18	7.51	3.27	3.53	0.63
Cryptophytes	Surface	288.1	110.5	11.1	4.16	28.27	14.21	898.7	268.2
	DCM	117.8	112.4	9.43	4.80	34.47	19.67	676.5	203.0
Diatoms	Surface	116.9	58.17	10.9	4.94	1983.9	1451	109.9	31.81
	DCM	272.2	102.2	56.82	50.46	2402	1321.2	139.4	33.33
Haptophytes	Surface	274.2	80.75	80.76	18.15	373.5	127.4	629.5	199.5
	DCM	225.7	50.45	85.85	41.23	369.5	150.8	583.1	151.1
PA	Surface	2.4	0.5	2.9	0.6	5.4	0.9	3.4	0.9
	DCM	2.6	0.5	3.6	1.4	5.2	1.0	3.3	0.5
PHP	Surface	0.46	0.17	0.31	0.18	1.04	0.44	0.40	0.13
	DCM	0.30	0.10	0.27	0.13	1.24	0.41	0.29	0.05
VA	Surface	4.5	2.0	3.0	1.5	21.5	10.7	13.5	4.1
	DCM	3.1	1.7	4.0	1.5	17.9	6.6	12.0	7.2
V1	Surface	2.4	1.1	1.6	0.8	8.7	4.1	6.8	2.1
	DCM	1.6	0.8	1.7	0.3	6.9	2.7	6.0	3.9
V2	Surface	1.8	0.8	1.2	0.7	10.8	5.9	6.1	2.0
	DCM	1.3	0.7	1.6	0.3	8.3	3.9	5.3	3.0
V3	Surface	0.3	0.1	0.2	0.1	2.2	1.2	0.7	0.3
	DCM	0.2	0.2	0.3	0.1	1.7	0.8	0.7	0.3

**Table 2:** Spearman correlation coefficients between total viral abundance (VA) and different viral abundance fractions (V1, V2, V3) with physicochemical and biological variables measured at surface (n = 47–50). Significant values after the Bonferroni–Holm correction (for a significance level of 0.05) are highlighted in bold. See Table 1 for units. Temp: temperature; Chl-a: chlorophyll a; PA: prokaryote abundance; PHP: prokaryotic heterotrophic production; Crypto: cryptophytes; Hapto: haptophytes; NO_3−_, NO_2−_, NH_4_^+^, SiO_4_^2−^, PO_4_, FDOM: fluorescent dissolved organic matter -peakT; DMSP: dimethyl sulfoniopropionate, DMS: dimethyl sulfide.

	Temp	Salinity	PA	Chl-a	PHP	Crypto.	Diatoms	Hapto.	NO_3−_	NO_2−_	NH_4_^+^	SiO_4_^2−^	PO_4_^3−^	FDOM	DMSP	DMS	Isoprene
VA	**0.78**	0.14	**0.50**	**0.78**	**0.53**	0.43	**0.78**	**0.65**	**−0.82**	0.38	−0.05	**−0.50**	**−0.70**	**0.47**	−0.18	**−0.53**	**0.78**
V1	**0.74**	0.14	**0.47**	**0.69**	0.41	0.38	**0.70**	**0.52**	**−0.67**	0.24	−0.08	**−0.51**	**−0.54**	0.39	−0.19	**−0.50**	**0.64**
V2	**0.78**	0.14	**0.54**	**0.78**	**0.50**	0.40	**0.77**	**0.64**	**−0.80**	0.35	−0.07	**−0.49**	**−0.70**	0.44	−0.21	**−0.51**	**0.74**
V3	**0.88**	0.31	**0.58**	**0.83**	**0.54**	0.41	**0.85**	**0.70**	**−0.82**	**0.50**	−0.11	**−0.50**	**−0.69**	0.43	−0.17	**−0.52**	**0.78**

## Supplementary Materials

The following are available online at https://www.mdpi.com/2075-1729/10/7/107/s1, Figure S1: Temperature and salinity profiles, Figure S2: Randomly amplified polymorphic DNA gel image, Figure S3: Viral community banding patter, Figure S4: NMDS of surface and DCM viral communities, Table S1: Physicochemical variables measured during the PEGASO cruise, Table S2: Biological variables measured during the PEGASO cruise.
