# Supplementary material for: Assessing Viral Abundance and Community Composition in Four Contrasting Regions of the Southern Ocean
Source: Life (Basel). 2020 Jul 5;10(7):107. doi: 10.3390/life10070107 (PMC7400478; doi:10.3390/life10070107)
Supplement: Supplementary file 1 [file life-10-00107-s001.pdf]

life

Supplementary Materials of Assessing Viral Abundance and Community Composition in Four Contrasting Regions of the Southern Ocean

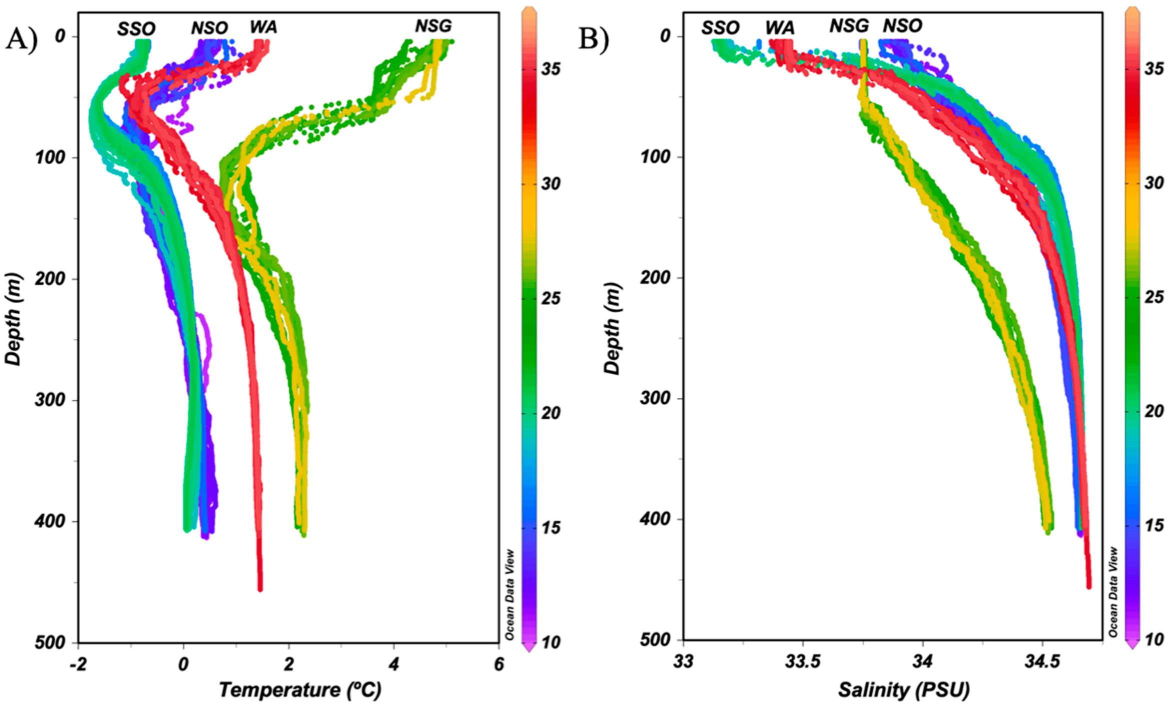

Figure S1. Temperature and salinity profiles. Profiles of (**A**) Temperature, and (**B**) Salinity from the four regions registered during the PEGASO expedition. The color indicates different sampling days. See the explanation in Figure 1 for acronyms.

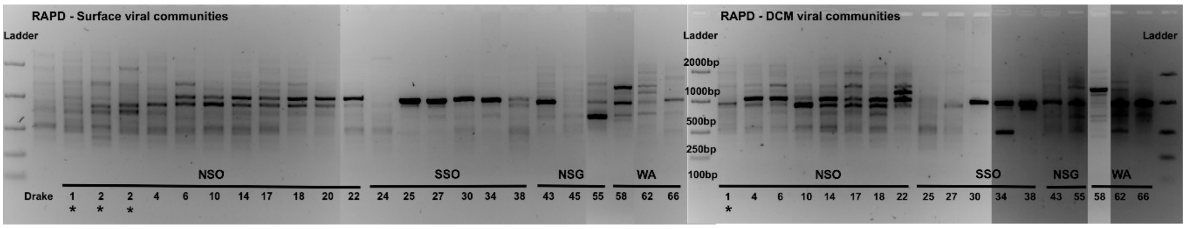

Figure S2. Randomly amplified polymorphic DNA gel image. Gel electrophoresis from viral DNA concentrates from the four regions and both depth layers (Surface and DCM). The numbers below each line indicate the station number. First, middle (indicated) and last lanes are the EasyLadder I (Ladder), Bioline. Molecular weight markers are indicated in base pairs (bp). Sharp differences on the gray shades are due to the side-merge of different gels images (several gels were run in order to work with the best quality gels). Asterisk (*) indicates that those lanes (i.e. stations) were not used in the statistical analyses. See the explanation in Figure 1 for acronyms.

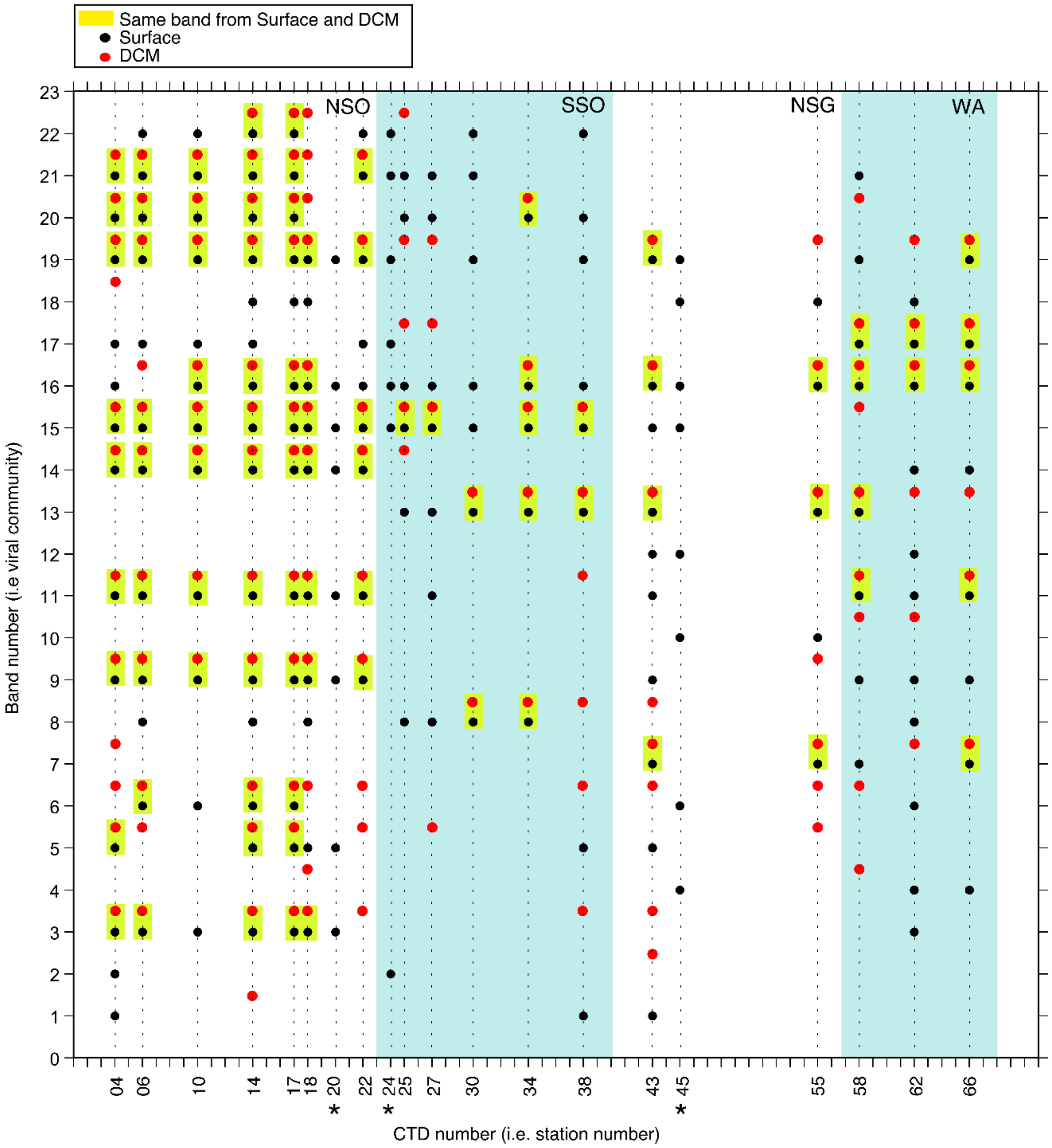

Figure S3. Viral community banding pattern. Summary of the band pattern obtained in the gel electrophoresis (Figure S2). The presence of bands at each station is depicted with a black dot (surface) and a red dot (DCM). When the same band showed up at both depth layers in the same station, a yellow box has been drawn. Asterisk (*) indicates the stations where no diversity samples were measured at the DCM. See the explanation in Figure 1 for acronyms.

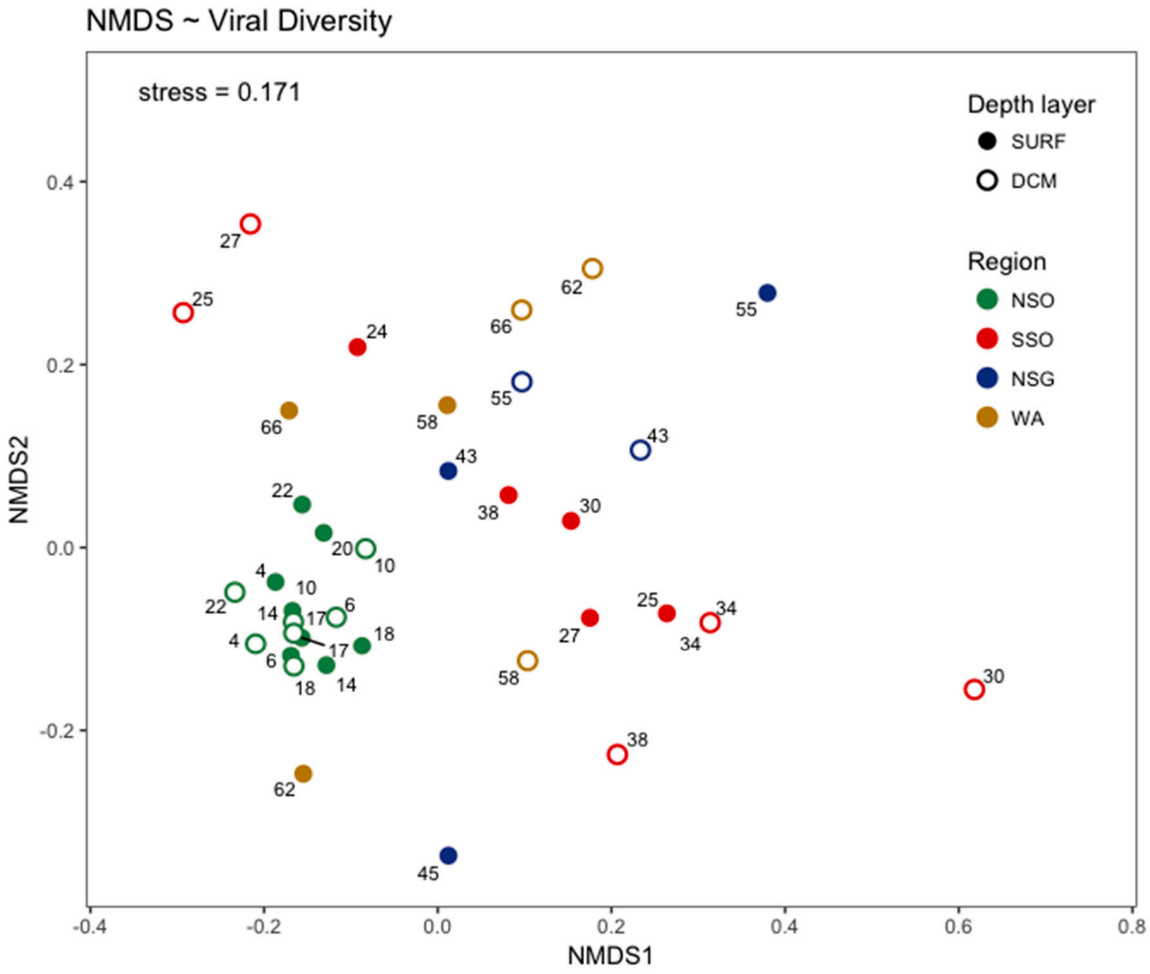

Figure S4. Non-Metric Multidimensional Scaling (NMDS) of the marine viral community composition from surface and DCM plotted together. Full dots represent surface communities and empty circles represent DCM samples. Numbers correspond to the station sampled. See explanation in Figure 1 for acronyms.

Table 1. Physicochemical variables measured during the PEGASO cruise. Sample code: station number and date. Lat (S): Latitude. Long (W): Longitude. Depth (m): surface -4m- and the DCM. Temperature, Salinity, Nutrients: nitrate (NO_3^-), nitrite (NO_2^-), ammonia (NH_4^+), silicate (SiO_4^{2-}), and phosphate (PO_4^{3-}). FDOM: Fluorescence of Dissolved Organic Matter peak-t. See explanation in Figure 1 for acronyms.

Sampling site	Sample code (PEGASO_station number_date[year/month/day])	Lat (S)	Long (W)	Depth (m)	Temperature (°C)	Salinity (PSU)	NO_3- (µM)	NO_2- (µM)	NH_4^+ (µM)	SiO_4^{2-} (µM)	PO_4^{3-} (µM)	FDOM (R.U)
NSO	PGS_CTD3_150110	600021	460876	4	0.51	33.89	28.215	0.184	0.209	47.05	1.45	22.29
NSO	PGS_CTD4_150110	600082	460402	4	0.57	33.83	29.979	0.231	1.369	45.77	2.17	17.48
		600082	460402	68	0.11	34.15	-	-	-	-	-	-
NSO	PGS_CTD6_150111	595873	460099	4	0.43	33.88	28.646	0.159	0.238	44.48	2.23	14.34
		595873	460099	45	0.65	34.08	-	-	-	-	-	-
NSO	PGS_CTD7_150111	595853	460268	4	0.58	33.90	27.022	0.164	0.571	44.90	2.12	15.31
		595853	460268	38	−0.36	33.99	-	-	-	-	-	-
NSO	PGS_CTD8_150111	595940	460092	4	0.53	33.90	26.705	0.197	0.795	43.01	2.00	2.20
		595940	460092	38	−0.19	33.99	-	-	-	-	-	-
NSO	PGS_CTD10_150111	595932	455931	4	0.53	33.89	26.257	0.288	6.56	43.81	2.00	2.27
		595932	455931	35	−0.01	34.00	-	-	-	-	-	-
NSO	PGS_CTD11_150112	595938	455824	4	0.45	33.91	28.975	0.209	1.342	48.99	2.08	-
		595938	455824	35	0.02	33.97	-	-	-	-	-	-
NSO	PGS_CTD12_150112	595931	455515	4	0.40	33.91	24.061	0.257	5.375	47.38	1.78	5.60
		595931	455515	36	−0.22	34.00	-	-	-	-	-	-
NSO	PGS_CTD14_150112	595937	455495	4	0.51	33.88	28.187	0.229	0.522	53.58	1.99	4.21
		595937	455495	38	0.01	34.00	-	-	-	-	-	-
NSO	PGS_CTD15_150112	595952	455504	4	0.79	33.91	25.49	0.222	0.884	51.72	1.88	3.96
		595952	455504	39	−0.33	34.03	-	-	-	-	-	-

NSO	PGS_CTD17_150112	600056	455265	4	0.86	34.09	29.96	0.195	0.367	55.51	2.23	3.82
		600056	455265	40	−0.37	33.99	-	-	-	-	-	-
NSO	PGS_CTD18_150113	600407	454605	4	0.84	33.88	27.837	0.206	0.795	47.36	2.08	3.63
		600407	454605	38	−0.20	33.99	-	-	-	-	-	-
NSO	PGS_CTD20_150114	600340	453974	4	0.45	33.74	28.871	0.242	1.74	48.11	2.17	3.43
		600340	453974	20	0.53	33.90	-	-	-	-	-	-
NSO	PGS_CTD21_150114	600367	454045	4	0.66	33.76	24.229	0.281	4.162	55.27	1.74	4.15
NSO	PGS_CTD22_150115	600402	454039	4	0.79	33.82	24.897	0.403	9.608	44.52	1.83	5.44
		600402	454039	35	0.18	34.01	-	-	-	-	-	-
NSO	PGS_CTD23_150115	600407	454099	4	0.91	33.85	-	-	-	-	-	-
SSO	PGS_CTD24_150116	611142	430133	4	−0.67	33.29	29.901	0.16	0.955	53.92	2.33	3.28
SSO	PGS_CTD25_150117	612236	434676	4	−0.88	33.13	28.975	0.152	1.027	54.37	2.15	5.26
		612236	434676	54	−1.48	34.08	-	-	-	-	-	-
SSO	PGS_CTD26_150118	611953	434708	4	−0.74	33.18	30.184	0.16	0.967	48.79	2.23	3.17
SSO	PGS_CTD27_150118	611839	435755	4	−0.84	33.19	25.841	0.144	0.862	44.87	1.92	4.21
		611839	435755	29	−1.06	33.84	-	-	-	-	-	-
SSO	PGS_CTD28_150118	611457	440023	4	-	-	32.232	0.152	0.974	45.72	2.46	3.14
SSO	PGS_CTD30_150119	611362	435427	4	−0.82	33.15	26.338	0.159	1.218	42.95	2.01	2.63
		611362	435427	44	−1.28	33.99	-	-	-	-	-	-
SSO	PGS_CTD31_150119	611378	435598	4	−0.78	33.14	27.141	0.16	1.564	46.86	2.08	3.65
		611378	435598	44	−1.49	34.09	-	-	-	-	-	-
SSO	PGS_CTD32_150119	611393	435424	4	−0.80	33.16	28.438	0.178	0.939	50.07	2.15	6.83
		611393	435424	75	−1.69	34.22	-	-	-	-	-	-
SSO	PGS_CTD34_150119	611342	435069	4	−0.78	33.15	17.691	0.17	2.492	36.20	1.44	3.75
		611342	435069	50	−1.51	34.10	-	-	-	-	-	-

SSO	PGS_CTD35_150120	611373	435519	4	−0.89	32.99	26.181	0.161	4.749	44.70	2.10	6.01
		611373	435519	40	−1.43	34.04	-	-	-	-	-	-
SSO	PGS_CTD36_150120	611531	435143	4	−0.93	33.10	27.332	0.167	1.552	50.04	2.15	3.10
		611531	435143	66	−1.62	34.23	-	-	-	-	-	-
SSO	PGS_CTD38_150120	611458	435347	4	−0.79	33.16	29.124	0.176	2.366	48.08	2.54	4.02
		611458	435347	51	−1.54	34.10	-	-	-	-	-	-
SSO	PGS_CTD39_150120	611416	435332	4	−0.79	33.15	28.789	0.185	1.362	49.72	2.15	4.55
		611416	435332	69	−1.62	34.21	-	-	-	-	-	-
SSO	PGS_CTD41_150120	611557	435446	4	−0.68	33.15	28.67	0.208	2.569	50.68	2.12	4.37
		611557	435446	69	−1.61	34.24	-	-	-	-	-	-
NSG	PGS_CTD43_150124	501475	395186	4	4.62	33.73	16.75	0.336	1.355	3.07	1.11	6.27
		501475	395186	13	4.24	33.76	-	-	-	-	-	-
NSG	PGS_CTD44_150124	501535	395168	4	5.19	33.76	16.578	0.236	0.771	2.48	1.41	7.72
		501535	395168	21	4.10	33.76	-	-	-	-	-	-
NSG	PGS_CTD45_150124	501625	394949	4	4.91	33.76	17.722	0.291	0.591	2.02	1.09	9.05
		501625	394949	21	4.07	33.76	-	-	-	-	-	-
NSG	PGS_CTD47_150125	501571	394578	4	4.82	33.75	17.155	0.303	0.781	1.51	1.03	6.35
		501571	394578	20	4.24	33.76	-	-	-	-	-	-
NSG	PGS_CTD48_150125	501549	394905	4	4.87	33.75	18.588	0.373	2.344	2.02	1.26	5.21
		501549	394905	25	4.67	33.76	-	-	-	-	-	-
NSG	PGS_CTD50_150125	501682	394604	4	4.86	33.75	14.651	0.273	0.753	1.84	1.18	7.00
		501682	394604	28	4.38	33.76	-	-	-	-	-	-
NSG	PGS_CTD52_150125	501626	394495	4	4.89	33.75	15.812	0.259	0.679	1.89	1.34	10.13
		501626	394495	48	4.16	33.76	-	-	-	-	-	-
NSG	PGS_CTD53_150125	501675	394522	4	4.94	33.75	15.121	0.247	0.36	1.96	1.29	

		501675	394522	15	4.47	33.76	-	-	-	-	-	-
NSG	PGS_CTD54_150125	501737	394555	4	5.02	33.75	15.925	0.264	0.792	1.73	1.22	10.80
		501737	394555	65	3.83	33.76	-	-	-	-	-	-
NSG	PGS_CTD55_150125	502082	394721	4	4.77	33.75	17.531	0.307	6.756	2.16	1.46	8.55
		502082	394721	66	3.37	33.79	-	-	-	-	-	-
NSG	PGS_CTD56_150125	502056	394914	4	4.95	33.75	16.403	0.304	5.208	2.02	1.47	5.78
		502056	394914	40	4.70	33.75	-	-	-	-	-	-
WA	PGS_CTD58_150202	643224	645319	4	1.38	33.39	18.483	0.185	4.421	49.75	1.74	4.92
		643224	645319	11	1.38	33.39	-	-	-	-	-	-
WA	PGS_CTD59_150202	643240	645186	4	1.46	33.38	17.006	0.18	2.243	46.18	1.70	6.70
		643240	645186	15	1.44	33.38	-	-	-	-	-	-
WA	PGS_CTD60_150202	643145	644885	4	1.49	33.39	18.698	0.232	2.706	54.79	1.51	7.17
		643145	644885	11	1.48	33.39	-	-	-	-	-	-
WA	PGS_CTD62_150202	643037	644964	4	1.41	33.42	19.25	0.167	2.226	54.93	1.88	6.95
		643037	644964	20	1.05	33.55	-	-	-	-	-	-
WA	PGS_CTD63_150203	643020	645361	4	1.33	33.42	18.574	0.16	2.946	51.15	1.66	9.53
		643020	645361	11	1.39	33.42	-	-	-	-	-	-
WA	PGS_CTD64_150203	642956	645046	4	1.38	33.44	19.042	0.247	7.528	45.02	1.81	9.99
		642956	645046	11	1.38	33.44	-	-	-	-	-	-
WA	PGS_CTD66_150203	642876	645159	4	1.40	33.45	18.63	0.199	1.702	45.67	1.83	15.63
		642876	645159	20	3.15	33.45	-	-	-	-	-	-
WA	PGS_CTD67_150203	642897	645494	4	1.48	33.43	18.299	0.201	2.484	49.01	1.99	10.62
		642897	645494	22	1.41	33.44	-	-	-	-	-	-
WA	PGS_CTD68_150203	642706	645344	4	1.57	33.44	20.374	0.156	1.485	50.67	2.02	14.30
		642706	645344	22	1.40	33.45	-	-	-	-	-	-

Table 2. Biological variables measured during the PEGASO cruise. Station code: station number and date. Depth (m): surface -4m- and the DCM. V1, V2, and V3: abundances of the different fluorescent-class fractions of the viral counts. Total VA: total viral abundance accounting for all fluorescent classes. VPR: Viral-Prokaryote ratio. Prokaryote Ab.: Prokaryote Abundance. PHP: Prokaryotic heterotrophic production. Chl-a: Chlorophyll a concentration. RAPD: indicates the samples from which analysis of fingerprinting (Randomly Amplified Polymorphic DNA) were performed. See explanation in Figure 1 for acronyms.

**Sampling site**	**Sample code (PEGASO_station number_date[year/month/day])**	**Depth (m)**	**V1 (10^6 mL^{-1})**	**V2 (10^6 mL^{-1})**	**V3 (10^6 mL^{-1})**	**Total VA (10^6 mL^{-1})**	**VPR**	**Prokaryote Ab. (10^5 mL^{-1})**	**PHP (μg C l^{-1}d^{-1})**	**Chl-a (μg L^{-1})**	**RAPD**
NSO	PGS_CTD3_150110	4	2.08	1.48	0.28	3.82	16.71	2.29	0.48	1.98	-
NSO	PGS_CTD4_150110	4	1.87	1.49	0.32	3.66	18.96	1.93	0.35	1.62	Yes
		68	1.07	1.03	0.19	2.27	7.72	2.94	0.27	0.92	Yes
NSO	PGS_CTD6_150111	4	4.27	3.46	0.31	7.94	28.80	2.76	0.81	1.81	Yes
		45	1.99	1.20	0.16	3.32	10.30	3.22	0.26	1.96	Yes
NSO	PGS_CTD7_150111	4	5.35	4.01	0.50	9.79	60.98	1.61	0.78	1.82	-
		38	2.01	1.68	0.29	3.95	23.18	1.70	0.36	1.75	-
NSO	PGS_CTD8_150111	4	4.23	2.97	0.38	7.51	44.32	1.70	0.75	1.93	-
		38	3.77	3.53	0.74	7.97	53.21	1.50	0.24	1.95	-
NSO	PGS_CTD10_150111	4	1.70	1.25	0.16	3.09	19.37	1.60	0.45	2.21	Yes
		35	1.63	1.13	0.20	2.94	15.45	1.90	0.32	1.95	Yes
NSO	PGS_CTD11_150112	4	2.66	1.98	0.36	4.96	16.84	2.95	0.46	2.07	-
		35	1.95	1.44	0.29	3.65	14.47	2.52	0.25	1.82	-
NSO	PGS_CTD12_150112	4	2.09	1.49	0.31	3.86	13.96	2.77	0.50	2.19	-
		36	1.30	1.18	0.24	2.70	8.95	3.02	0.26	1.43	-
NSO	PGS_CTD14_150112	4	1.39	1.81	0.29	3.47	13.76	2.52	0.46	1.93	Yes
		38	1.07	0.97	0.20	2.23	8.47	2.63	0.15	1.67	Yes
NSO	PGS_CTD15_150112	4	1.14	0.83	0.17	2.12	9.21	2.30	0.36	1.58	-
		39	0.20	0.19	0.02	0.41	1.52	2.67	0.31	1.17	-
NSO	PGS_CTD17_150112	4	2.26	1.70	0.27	4.20	18.24	2.30	0.39	2.11	Yes

		40	0.18	0.19	0.02	0.39	1.55	2.50	0.24	0.78	Yes
NSO	PGS_CTD18_150113	4	1.74	1.17	0.23	3.11	10.93	2.85	0.62	2.05	Yes
		38	1.19	0.94	0.12	2.23	0.00	0.00	0.34	1.39	-
NSO	PGS_CTD20_150114	4	1.76	1.18	0.19	3.11	14.25	2.18	0.50	1.34	Yes
		20	2.44	1.38	0.23	4.02	13.62	2.95	0.60	1.87	Yes
NSO	PGS_CTD21_150114	4	1.58	1.26	0.24	3.06	12.83	2.39	0.24	1.96	-
NSO	PGS_CTD22_150115	4	2.97	1.93	0.33	5.20	22.36	2.32	0.47	1.72	Yes
		35	1.39	0.91	0.17	2.45	9.45	2.59	0.28	1.78	Yes
NSO	PGS_CTD23_150115	4	1.68	1.58	0.19	3.42	-	-	-	1.82	-
SSO	PGS_CTD24_150116	4	0.46	0.63	0.09	1.18	5.17	2.27	0.89	0.28	Yes
		46	-	-	-	-	-	-	-	0.38	-
SSO	PGS_CTD25_150117	4	1.81	1.58	0.17	3.53	13.92	2.53	0.37	0.34	Yes
		54	1.59	1.79	0.23	3.60	8.27	4.35	0.64	0.36	Yes
SSO	PGS_CTD26_150118	4	0.91	1.14	0.16	2.20	9.08	2.42	0.25	0.36	-
SSO	PGS_CTD27_150118	4	1.14	1.26	0.10	2.48	6.86	3.62	0.33	0.45	Yes
		29	1.29	1.39	0.21	2.86	6.49	4.41	1.00	0.44	-
SSO	PGS_CTD28_150118	4	19.16	1.39	0.79	1.17	3.90	3.00	0.08	0.45	-
		70	-	-	-	-	-	-	-	0.29	-
SSO	PGS_CTD30_150119	4	3.40	2.98	0.27	6.59	24.97	2.64	0.35	0.28	Yes
		44	3.02	2.72	0.54	6.23	16.00	3.89	0.57	0.31	-
SSO	PGS_CTD31_150119	4	1.09	0.95	0.16	2.18	8.79	2.48	0.27	0.30	-
		44	1.56	1.62	0.32	3.47	9.06	3.84	0.25	0.84	-
SSO	PGS_CTD32_150119	4	1.70	1.33	0.16	3.17	13.73	2.31	0.17	0.29	-
		75	2.02	2.19	0.34	4.51	27.09	1.66	0.14	0.66	-
SSO	PGS_CTD34_150119	4	2.90	2.14	0.18	5.17	21.95	2.36	0.44	0.29	Yes

		50	1.37	1.40	0.22	2.97	6.35	4.67	0.17	0.32	Yes
SSO	PGS_CTD35_150120	4	2.59	1.38	0.13	4.06	13.73	2.96	0.22	0.29	-
		40	2.00	1.72	0.29	3.98	8.07	4.93	0.10	0.31	-
SSO	PGS_CTD36_150120	4	1.71	1.21	0.18	3.08	11.01	2.80	0.19	0.28	-
		66	1.30	1.43	0.32	3.03	11.31	2.68	0.29	0.48	-
SSO	PGS_CTD38_150120	4	1.17	0.75	0.11	2.02	4.05	4.98	0.27	0.28	Yes
		51	1.02	0.87	0.18	2.06	4.67	4.40	0.29	0.59	-
SSO	PGS_CTD39_150120	4	1.26	1.22	0.22	2.69	8.57	3.14	0.37	0.28	-
		69	1.59	1.27	0.28	3.12	8.30	3.75	0.52	0.61	-
SSO	PGS_CTD41_150120	4	0.88	0.65	0.13	1.65	5.12	3.22	0.20	0.30	-
		69	1.36	1.07	0.20	2.61	8.92	2.93	0.00	0.45	-
NSG	PGS_CTD43_150124	4	9.00	7.40	1.48	17.77	24.59	7.23	0.94	8.95	Yes
		13	4.76	6.90	1.60	13.22	21.69	6.10	0.93	9.65	Yes
NSG	PGS_CTD44_150124	4	6.75	6.92	1.60	15.17	32.88	4.61	0.53	4.55	-
		21	5.16	6.85	2.03	13.98	30.22	4.63	0.81	14.72	-
NSG	PGS_CTD45_150124	4	3.96	5.51	1.66	11.10	26.13	4.25	0.95	5.74	Yes
		21	5.28	6.87	1.92	14.01	32.86	4.26	0.85	10.07	-
NSG	PGS_CTD47_150125	4	7.42	7.17	1.36	15.85	37.10	4.27	1.08	6.69	-
		20	8.34	7.54	1.55	17.34	39.89	4.35	1.15	9.55	-
NSG	PGS_CTD48_150125	4	6.40	5.57	1.07	12.96	22.59	5.74	0.89	3.75	-
		25	4.00	3.58	0.84	8.38	16.06	5.22	0.90	6.17	-
NSG	PGS_CTD50_150125	4	17.49	24.06	4.58	45.89	87.50	5.24	0.54	5.62	-
		28	11.63	15.71	3.40	30.58	86.97	3.52	0.63	8.40	-
NSG	PGS_CTD52_150125	4	9.62	17.36	4.23	31.10	59.94	5.19	1.25	5.41	-
		48	10.43	13.56	2.66	26.52	53.81	4.93	1.88	7.35	-
NSG	PGS_CTD53_150125	4	13.71	15.01	2.12	30.59	62.75	4.87	1.73	5.45	-

		15	11.17	13.86	2.21	27.33	53.81	5.08	1.58	6.77	-
NSG	PGS_CTD54_150125	4	11.82	15.55	3.22	30.38	55.32	5.49	1.94	2.18	-
		65	5.32	5.51	1.39	12.17	20.63	5.90	1.98	3.53	-
NSG	PGS_CTD55_150125	4	4.09	7.31	1.62	12.95	20.44	6.33	1.07	5.31	-
		66	5.16	5.43	0.89	11.40	17.14	6.65	1.72	6.06	Yes
NSG	PGS_CTD56_150125	4	4.86	6.90	0.96	12.65	19.48	6.50	0.57	1.92	-
		40	5.31	5.75	0.61	11.62	15.94	7.29	0.95	3.53	-
WA	PGS_CTD58_150202	4	6.37	6.03	0.65	12.96	30.89	4.19	0.49	4.91	-
		11	5.46	4.99	0.57	10.94	47.74	2.29	0.38	5.30	-
WA	PGS_CTD59_150202	4	8.54	7.32	0.45	16.14	68.18	2.37	0.37	3.65	-
		15	4.80	4.03	0.59	9.35	33.30	2.81	0.28	4.31	-
WA	PGS_CTD60_150202	4	4.05	4.70	0.64	9.31	24.72	3.77	0.32	4.61	-
		11	3.59	4.06	0.57	8.15	22.08	3.69	0.26	4.38	-
WA	PGS_CTD62_150202	4	3.26	4.22	0.59	8.03	21.58	3.72	0.27	3.91	Yes
		20	3.38	4.57	0.74	8.64	16.43	5.26	0.14	1.97	Yes
WA	PGS_CTD63_150203	4	6.77	7.57	0.97	15.18	59.80	2.54	0.13	3.41	-
		11	1.36	1.71	0.23	3.28	12.31	2.66	0.15	3.24	-
WA	PGS_CTD64_150203	4	6.71	4.90	0.87	12.40	48.71	2.55	0.35	4.28	-
		11	5.95	4.87	0.65	11.37	46.42	2.45	0.31	3.93	-
WA	PGS_CTD66_150203	4	7.16	5.30	0.59	12.93	54.09	2.39	0.34	4.14	Yes
		20	8.13	5.67	1.06	14.77	59.93	2.47	0.30	3.97	Yes
WA	PGS_CTD67_150203	4	6.98	4.47	0.21	11.50	24,86	4.64	0.43	3.90	-
		22	-	-	-	-	-	4.66	0.32	2.66	-
WA	PGS_CTD68_150203	4	11.03	10.72	1.16	22.72	52.24	4.35	0.52	3.67	-
		22	15.16	12.79	1.36	29.09	66.25	4.39	0.28	3.64	-

© 2020 by the authors. Submitted for possible open access publication under the terms and conditions of the Creative Commons Attribution (CC BY) license (http://creativecommons.org/licenses/by/4.0/).
